# Pencil It in: Exploring the Feasibility of Hand-Drawn Pencil Electrochemical Sensors and Their Direct Comparison to Screen-Printed Electrodes

**DOI:** 10.3390/bios6030045

**Published:** 2016-08-29

**Authors:** Elena Bernalte, Christopher W. Foster, Dale A.C. Brownson, Morgane Mosna, Graham C. Smith, Craig E. Banks

**Affiliations:** 1 Faculty of Science and Engineering, Manchester Metropolitan University, Chester Street, Manchester M1 5GD, UK; ebernaltemorgado@gmail.com (E.B.); chris.w.foster@mmu.ac.uk (C.W.F.); d.brownson@mmu.ac.uk (D.A.C.B.); morgane.mosna@etu.iut-tlse3.fr (M.M.); 2 Departamento de Química Analítica e IACYS, Facultad de Ciencias, Universidad de Extremadura, Avda. de Elvas s/n, 06006 Badajoz, Spain; 3 Faculty of Science and Engineering, Department of Natural Sciences, University of Chester, Thorntorn Science Park, Pool Lane, Ince, Manchester CH2 4NU, UK; graham.smith@chester.ac.uk

**Keywords:** pencil-drawn electrodes, screen-printed electrodes, sensors, electrochemistry

## Abstract

We explore the fabrication, physicochemical characterisation (SEM, Raman, EDX and XPS) and electrochemical application of hand-drawn pencil electrodes (PDEs) upon an ultra-flexible polyester substrate; investigating the number of draws (used for their fabrication), the pencil grade utilised (HB to 9B) and the electrochemical properties of an array of batches (i.e, pencil boxes). Electrochemical characterisation of the PDEs, using different batches of HB grade pencils, is undertaken using several inner- and outer-sphere redox probes and is critically compared to screen-printed electrodes (SPEs). Proof-of-concept is demonstrated for the electrochemical sensing of dopamine and acetaminophen using PDEs, which are found to exhibit competitive limits of detection (3σ) upon comparison to SPEs. Nonetheless, it is important to note that a clear lack of reproducibility was demonstrated when utilising these PDEs fabricated using the HB pencils from different batches. We also explore the suitability and feasibility of a pencil-drawn reference electrode compared to screen-printed alternatives, to see if one can draw the entire sensing platform. This article reports a critical assessment of these PDEs against that of its screen-printed competitors, questioning the overall feasibility of PDEs’ implementation as a sensing platform.

## 1. Introduction

Among academia and industry, there is constant focus on the creation of low cost and efficient analytical techniques. In consideration of this, electrochemically-based analytical systems have been continually analysed and benchmarked. Indeed, the development of portable, low cost, and miniaturised analytical devices has promoted a true scientific revolution over the last decades [1].

The utilisation of “popular’’ carbon-based materials offers exciting possibilities within such electrochemical devices in general, due to their cost-effective production, that can exhibit similar or enhanced performance to that of the traditional noble metal based alternatives. An extremely attractive and effective technique to incorporate these electroactive materials is via the utilisation of screen-printing technology [2]. These screen-printed sensors have actually transformed the field due to their capability to bridge the gap between laboratory experiments with in-field implementation [3,4,5,6,7]. This is exemplified by the billions of dollars (per annum) that the glucose sensing market has benefited from in its use of screen-printed electrodes, as these handheld sensors allow individuals to measure their own blood glucose levels in the comfort of their home [8,9]. Such technology allows for the mass production of highly reproducible electrode configurations and, due to scales of economy, inexpensive sensing and disposable electrochemical platforms can be regularly fabricated [3].

**Figure 1:**
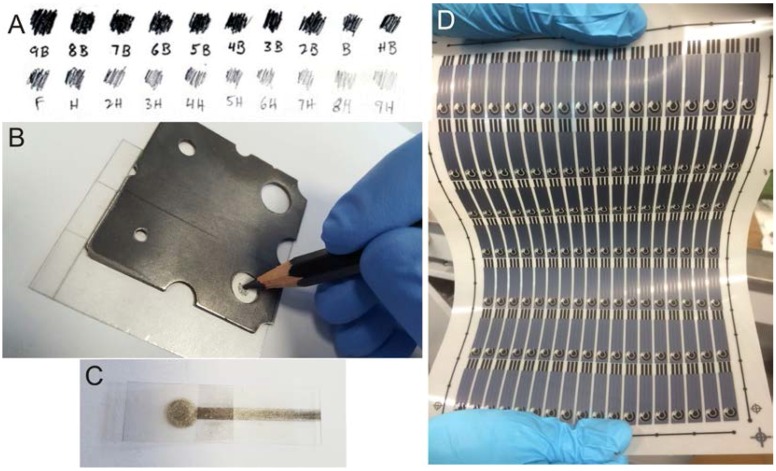
Different pencil grades and their graphite deposition (**A**); fabrication of Pencil-drawn Electrodes (**B**); a typical final Pencil-drawn Electrode (**C**) and an image of a sheet of screen-printed electrodes fabricated via the screen-printing process (**D**).

However, as electrochemists, we are constantly searching for novel electrode configurations. Taking advantage of pencil-based approaches that have just begun [10], we now focus on the creation of readily available hand-drawn pencil graphitic electrodes (PDEs), where one can potentially draw their own electrochemical system. The pencil-drawn approach offers an interesting method to develop sensing platforms, where devices can be fabricated in minutes using nothing more than readily available pencils. A pencil’s ability to support these platforms is heavily reliant upon the transfer of the graphitic material comprising it to the substrate. As is well known, standard pencils are “graded’’ based on the hardness of their leads, and pencils are classified on a scale from 9H to 9B, as presented in Figure 1A. The difference in blackness arises from the different relative fractions of graphite in the composition between harder and softer pencil leads. Recently, we have reported this intriguing concept where the utilisation of a PDE drawn 10 times with a 6B Staedtler pencil was shown to be an advantageous electrochemical platform, in terms of electrochemical reversibility and peak height/analytical signal during its electrochemical characterisation [11]. Upon its application, it was observed that in the majority of cases, electrochemical oxidation of an array of analytes was not feasible using these PDEs, unless a prior electrochemical reduction step was first implemented.

As mentioned previously, this is an area that has received increased interest over recent years, and Table 1 provides an overview of the current research into the electroanalytical application of PDEs. For example, Dossi et al. [17] studied the performance of PDEs on paper substrates for the detection of ascorbic acid, with additional work utilising cobalt(II) phthalocyanine doped-PDEs, where the cobalt(II) phthalocyanine was mixed with the bulk pencil “lead’’, sodium bentonite and potassium silicate mixture, and placed within a similar pencil setup and explored for the electrocatalytic detection of cysteine and hydrogen peroxide [17]. Additional reports from this group have explored the electrochemical detection of analytes such as potassium ferrocyanide [12], 1,2-hydroxybenzene [14], dopamine, paracetamol [13] and ortho-diphenols in edible oil samples [19]. Also, these PDEs have been recently implemented in electrophoresis devices for an interesting contactless conductivity detection of inorganic cations in human tears [1]. Honeychurch has demonstrated that the electrochemical detection of lead(II) (within real canal water samples) can also be achieved via the use of hand-drawn PDEs on a polyvinylchloride substrate [18]. Furthermore, fully-drawn electroanalytical sensors in a different configuration have been reported by Li et al. for point-of-care applications, involving the determination of dopamine [20] and glucose [21].

In this paper, we critically analyse pencil-drawn electrodes (PDEs) that have been fabricated with a range of commercially available Derwent pencils. We compare the electron transfer properties and electrochemical sensing capabilities for the detection of dopamine and acetaminophen of our hand-drawn electrodes to that of graphitic-based screen-printed electrodes. In addition, we analyse the effect of a pencil-drawn reference electrode and compare it to screen-printed alternatives, exploring the overall feasibility and suitability of these PDEs as a full electrode system.

## 2. Materials and Methods 

### 2.1. Chemicals

All chemicals were purchased from Sigma-Aldrich at analytical grade and were used as received without any further purification. The solutions were prepared with deionised water of resistivity not less than 18.2 MΩ·cm (25 °C) containing 0.1 M KCl supporting electrolyte and were thoroughly degassed with nitrogen to remove oxygen before analysis when required. 1 mM stock solutions of acetaminophen and dopamine were prepared separately in 0.1 M pH 7.4 phosphate buffer solutions (PBS) and kept in the fridge until assayed.

### 2.2. Instrumentation

Electrochemical measurements were carried out with a Palmsens (Palm Instruments BV, Houten, The Netherlands) and a µ-Autolab III (ECO-Chemie, Utrecht, The Netherlands) potentiostats. All experiments throughout this study were conducted using a three electrodes configuration utilising nickel coil and Saturated Calomel Electrodes (SCE) as a counter and reference, respectively. Scanning electron microscope (SEM) images and surface element analysis were obtained with a Supra 40VP model SEM (Carl Zeiss Ltd., Cambridge, UK) coupled to an Apollo 40 SDD energy-dispersive X-ray (EDX) microscope (EDAX, Cambridge, UK). Raman Spectroscopy was performed using a “inVia’’ confocal Raman Microscope (Renishaw PLC, York, UK) equipped with a confocal microscope (×50 objective) spectrometer with an argon laser (514 nm excitation) and a very low laser power level (0.8 mW) to avoid any heating effects. The XPS data was acquired using a bespoke ultra-high vacuum system fitted with a Specs GmbH Focus 500 monochromated Al Kα X-ray source, Specs GmbH Phoibos 150 mm mean radius hemispherical analyser with 9-channeltron detection, and a Specs GmbH FG20 charge neutralising electron gun [22]. Survey spectra were acquired over the binding energy range 1100–0 eV using a pass energy of 50 eV and high resolution scans were made over the C 1s and O 1s lines using a pass energy of 20 eV. In each case, the analysis was an area-average over a region approximately 1.4 mm in diameter on the sample surface, using the 7 mm diameter aperture and lens magnification of ×5. The energy scale of the instrument is calibrated according to ISO 15472, and the intensity scale is calibrated using an in-house method traceable to the UK National Physical Laboratory [23]. Data were quantified using Scofield cross sections corrected for the energy dependencies of the electron attenuation lengths and the instrument transmission [24]. Data interpretation was carried out using CasaXPS software v2.3.16 [25]. Electrical resistance measurements were carried out using a 4-wire measurement method, usually used to measure small resistances in thin films. The benefit of this system is that it prevents the resistance in the wires and connectors from being included in any measurements. 

### 2.3. Design and Fabrication of the Electrodes

The pencil-drawn electrodes (denoted as PDEs herein) were used as a working electrodes and were fabricated by systematic hand-tracing of an 8 mm diameter circle using a custom-made/fabricated stainless steel template to define the area onto a flexible polyester substrate (Autotex AM, model F157L, 150 µm thickness); this is shown in Figure 1B,C. Commercially available HB grade soft graphite pencils (Graphic 12, Derwent, Workington, UK) from six different boxes were used for the fabrication of several PDEs. Upon referring to “one draw’’ within this paper, this stipulates that we have moved the pencil whilst in contact with the substrate such that the complete area within the 8 mm diameter circle/disc (to be defined as the working area) is drawn as shown in Figure 1. After defining the surface area, a connecting strip from the top of the circle allows for a crocodile clip connection to be employed with the potentiostat [26]. It is noted that a prior report indicated that these polyester based electrodes do not suffer from capillary action as observed in the case of paper-based sensors, causing the solution to wick-up the electrode towards the electrical connections and resulting in electrical shorting, thus compromising the electrochemical measurement [27].

Screen-printed graphite macroelectrodes (SPEs) were also used for comparative purposes. SPEs, which have a 3 mm diameter working electrode, were fabricated in-house with appropriate stencil designs using a DEK 248 screen-printing machine (DEK, Weymouth, UK) (Figure 1D). A previously used carbon-graphite ink formulation (product code: C2000802P2; Gwent Electronic Materials Ltd., Pontypool, UK) was first screen-printed onto a polyester flexible film (Autostat, 250 µm thickness). This layer was cured in a fan oven at 60 °C for 30 min. Next, a silver/silver chloride pseudo reference electrode was included by screen-printing Ag/AgCl paste (product code: C2030812P3; Gwent Electronic Materials Ltd., Pontypool, UK) onto the polyester substrate. A dielectric paste/ink (product code: D2070423D5; Gwent Electronic Materials Ltd., Pontypool, UK) was next printed to cover the connections. After curing again at the same conditions as before, the screen-printed electrodes were ready to be used. The SPEs were then precisely cut to remove the Ag/AgCl pseudo reference and carbon counter, and used as part of a standard three electrode configuration within our electrochemical cell.

### 2.4. Determination of the Heterogeneous Electron Transfer Rate Kinetics (k^0^) of the PDEs

The *k*^0^ values for the PDEs were deduced using the Nicholson equation for an electrochemically *quasi*-reversible process as described by Equation (1):

(1) $$\varphi =k^{0}{\left[\pi Dn\upsilon F/\left(RT\right)\right]}^{-1/2}$$

where ϕ is a dimensionless kinetic parameter, *D* is the diffusion coefficient (9.1 × 10^−6^ cm^2^·s^−1^ for hexaammineruthenium (III) chloride) [28], *n* is the number of electrons involved in the electrochemical process, *F* is the Faraday constant, ʋ is the voltammetric scan rate, *R* is the universal gas constant, and *T* is the temperature of the solution. The kinetic parameter, ϕ, is tabulated as a function of peak-to-peak separation (*ΔEp*) at a set of temperature (298 K) for a one-step, one electron process (where α = 0.5). The function of ϕ (*ΔEp*), which fits Nicholson data for practical usage (rather than producing a working curve) is given by Equation (2):

(2) φ=(−0.628+0.0021X)/(1−0.017X)

where *X* = *ΔEp*, is used to determine ϕ as a function of *ΔEp* from the experimentally recorded voltammetry. From this, a plot of ϕ against ${\left[\pi Dn\upsilon F/\left(RT\right)\right]}^{-1/2}$ is produced graphically allowing the *k*^0^ to readily determined. However, for *ΔEp* values that exceed 212 mV within the Nicholson table, note that one has to rely upon Equation (3):

(3) $$\varphi =\left[2.18(D\alpha nF\upsilon /{\left(RT\right)}^{0.5}\right]exp^{\left[-\left(\left(\alpha^{2}nF\right)/RT\right)x\Delta Ep\right]}$$

where α is assumed to correspond to 0.5.

## 3. Results and Discussion

In this paper, we report the electrochemical and physiochemical characterisation of pencil-drawn electrodes (PDEs) fabricated on ultra-flexible polyester substrates. These PDEs are evaluated in terms of pencil “batch’’ reproducibility (i.e., pencils from different boxes) and the overall feasibility of these electrode systems in terms of electrochemical sensing in comparison to commonly utilised screen-printed electrodes (SPEs), considering aspects such as the pencil grade used for the fabrication, analytical sensitivity and other surface features.

### 3.1. Electrochemical Characterisation of Pencil-Drawn Electrodes (PDEs)

#### 3.1.1 Optimisation of the Number of Draws

**Figure 2:**
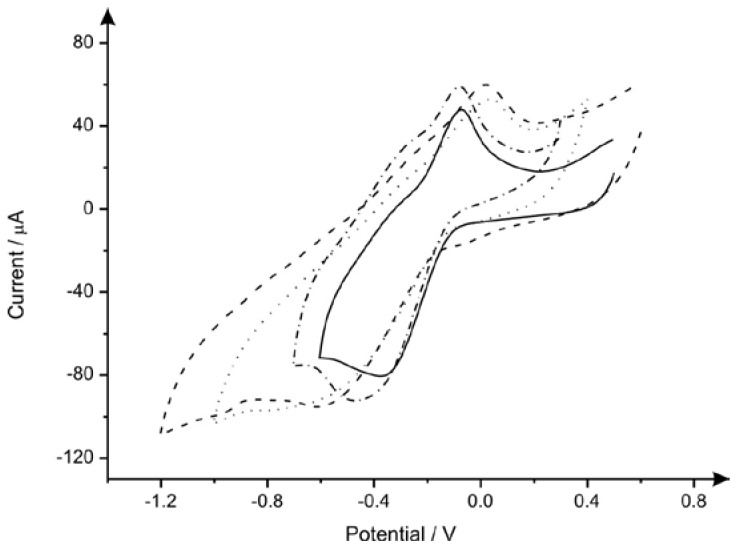
Typical cyclic voltammograms recorded with 1 mM hexaammineruthenium(III) chloride/0.1 M KCl using PDE1 (HB pencil, Box 1) drawn 15 (dotted line), 30 (dashed line), 60 (solid line) and 100 (dashed dotted line) times. Scan rate: 50 mV·s^−^^1^.

We first optimise the amount of graphite deposited onto the polyester substrate by hand-drawing 15, 30, 60 and 100 times (see Figure 1) through comparison of its electrochemical performance using the outer-sphere redox probe hexaammineruthenium (III) chloride. The pencil grade “HB’’ was the first chosen from Box 1 (the corresponding electrode fabricated was named as PDE1 throughout) with the effect of the number of draws, and consequently the quantity of graphite transferred onto the polyester support being examined. This was benchmarked through the determination of the standard heterogeneous rate constant (*k*^0^) calculated using Equations (1)–(3) over a range of voltammetric scan rates (5–1000 mV·s^−1^), utilising PDEs (from PDE1) that have been drawn 15, 30, 60 and 100 times, with values found to correspond to 8.81 × 10^−5^ cm·s^−1^, 8.38 × 10^−5^ cm·s^−1^, 3.53 × 10^−4^ cm·s^−1^ and 3.28 × 10^−4^ cm·s^−1^, respectively. As can be observed, a general increase of *k*^0^ values is evident. However, upon utilising a PDE drawn 100 times, there is no further significant improvement among the value for *k*^0^. Consequently, 60 times was chosen for further experiments. A plot of peak height vs. the square root of scan rate was performed and analysed using the outer-sphere redox probe, hexaammineruthenium (III) chloride, which indicates that such reaction is a diffusional controlled process, with a notably clear linear depiction (*R*^2^ = 0.9323) exhibited by 60 PDEs. Next, analysis of the cyclic voltammetric profiles of the same PDE1 “pencilled in’’ 15, 30, 60 and 100 times (Figure 2) revealed a noteworthy low value for the voltammetric peak-to-peak separation (*ΔEp* 254 mV (vs. SCE)) when using PDE1 drawn 60 times. The PDE1s drawn 15 and 30 times have values that correspond to 493 mV and 595 mV respectively, indicating that an improvement within the reversibility is offered when utilising PDEs that have been drawn an increased number of times. Intriguingly, this occurrence is not presented when using a PDE that has been drawn 100 times, where the value for the peak-to-peak separation is 360 mV, possibly due to the graphite’s adherence to the underlying substrate. 

#### 3.1.2. Influence of Different Pencil Grades upon the Fabrication of PDEs

As mentioned previously, the composition of pencils’ lead is a mixture of graphite, clay and wax. The combination of these components in different proportions determines the grade of the pencil and gives rise to distinct properties. Higher grades are associated with an increasing amount of graphite within the pencils, making them softer and darker when drawn on a substrate. In order to examine the influence of the different grades used for the fabrication of PDEs, the electrochemical response of various PDEs were evaluated using the outer-sphere redox probe hexaammineruthenium(III) chloride in 0.1 M KCl solution.

**Figure S1:**
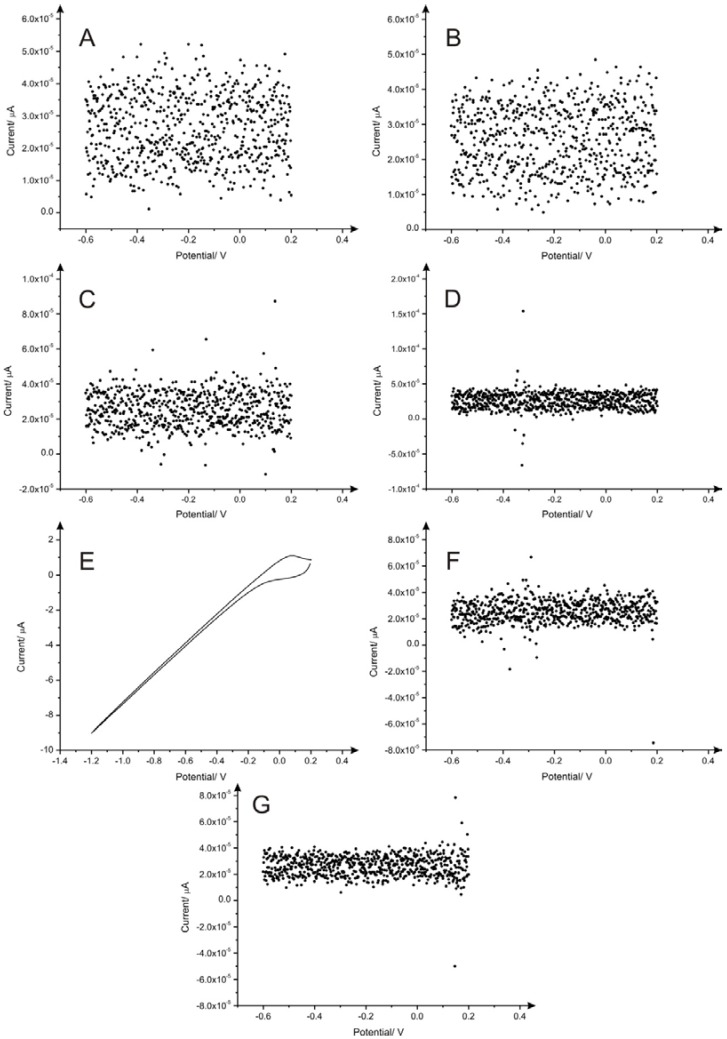
Cyclic voltammograms of 60 times drawn PDEs fabricated applying different pencil grades B (**A**); 2B (**B**); 3B (**C**); 4B (**D**); 7B (**E**), 8B (**F**) and 9B (**G**) from Box 1. Redox probe: hexaammineruthenium(III) chloride/0.1 M KCl. Scan rate: 5 mV·s^−1^.

Following the procedure described in the Materials and Methods Section, different PDEs were fabricated (applying 60 draws) using a B, 2B, 3B, 4B, 7B, 8B and 9B grades of pencils from the designated Box 1. Figure S1 illustrates typical cyclic voltammograms (CVs) obtained for different grades of PDEs. Unexpectedly, besides the PDE fabricated using pencil 7B, which reported a very resistive CV towards hexaammineruthenium(III) chloride redox probe, none of the other PDEs provided effective electrochemical responses, possibly due to the combination of graphite and clay within the pencil lead not creating an adequate conductive layer on the polyester substrate. It is important to note that the subsequent utilisation of higher pencil grades did not provide the expected shades when drawn on the plastic substrate and all the pencil leads (i.e., B, 2B, 3B, 4B, 7B, 8B and 9B) were surprisingly fragile.

**Figure S2:**
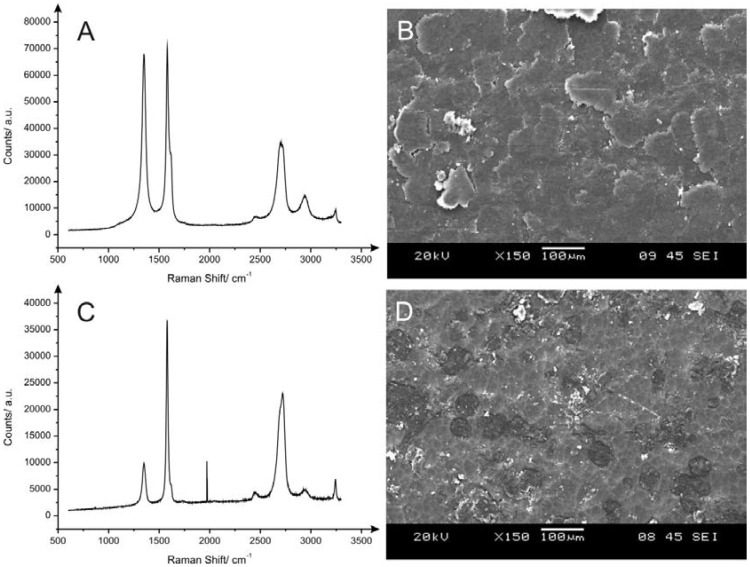
Raman spectra and SEM images of PDEs fabricated using HB (**A**) and (**B**) and 4B (**C**) and (**D**) pencils from Box 1.

Raman spectroscopy and SEM analysis of PDEs fabricated using pencil HB and 4B from Box 1 were carried out to investigate any correlation between the electrochemical behaviour of different grade PDEs and the morphology and characteristics of their surfaces. Figure S2A,C shows typical a Raman spectra obtained for the HB and 4B hand-drawn pencil leads. Interestingly, comparison of the characteristic spectra shows thicker and higher quality graphite in the 4B pencil when compared to the HB alternative, which is indicated by the 2D/G peak ratios evident at 2700 cm^–1^ (2D band) and 1550 cm^–1^ (G band), respectively [29,30]. Also evident is the slight protrusion (shoulder) present on the 2D peak in the case of the 4B pencil, supporting the former statement. Regarding the oxygenated species (or degree of edge plane defects) present, a D peak (1300 cm^–1^) appears in both samples, with a less intense peak present in the 4B pencil lead suggesting a lower level of oxygenated species content (or a smaller coverage of edge plane like- sites/defect sites) in this case compared to that of the HB. Thus, the none conductive nature of the 4B likely results from other sources within the 4B pencil lead, other/rather than being due to the quality of graphite present. We infer this is due to adhesive considerations with the underlying surface and influences of clay content, further, with higher levels of edge plane like- sites/defects present on the HB pencil likely leading to faster and more reactive electrochemical charge transfer occurring in that case. The latter point concerning the adhesive considerations of the pencil lead onto the substrate is also illustrated in Figure S2B, with SEM images showing a dense and graphitic deposition upon using the HB pencil. The graphite deposit utilising the 4B is vastly contrasted to that of the HB and it is clear that less material and lower quality graphite has adhered to the surface in this case (Figure S2D). Whatever the reason, this phenomenon is highly interesting and will require separate, more independently focused studies to fully understand the mechanism, which surpasses the intended scope of this paper. For the purpose of this work, we can conclude that the HB pencil performs more favourably than the 4B and other grades; thus, for further tests we focus solely on the use of this pencil type.

### 3.2. Reproducibility of Pencil Batches: Physicochemical and Electrochemical Characterisation

Next, our attention was turned to investigating the reproducibility of PDEs when employing the fabrication of five other batches of HB pencils belonging to the same commercial brand, as reported in the Experimental Section. Following the same procedure described throughout this paper, it draws were performed 60 times on the polyester substrate for the fabrication of new PDEs, consecutively designated as PDE2, PDE3, PDE4, PDE5 and PDE6 (from boxes 2 to 6, respectively).

**Figure S3:**
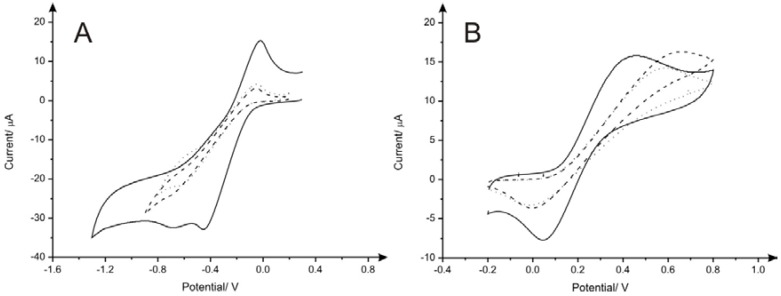
Comparison of cyclic voltammograms recorded for PDEs fabricated using HB pencils from boxes 1, 3 and 5, respectively, in 1 mM hexaammineruthenium(III) chloride (**A**) and 1 mM potassium ferrocyanide(II) (**B**) redox probes/0.1 M KCl. Scan rate: 5 mV·s^−1^. PDE1 (solid line), PDE3 (dashed line) and PDE5 (dotted line).

**Figure S4:**
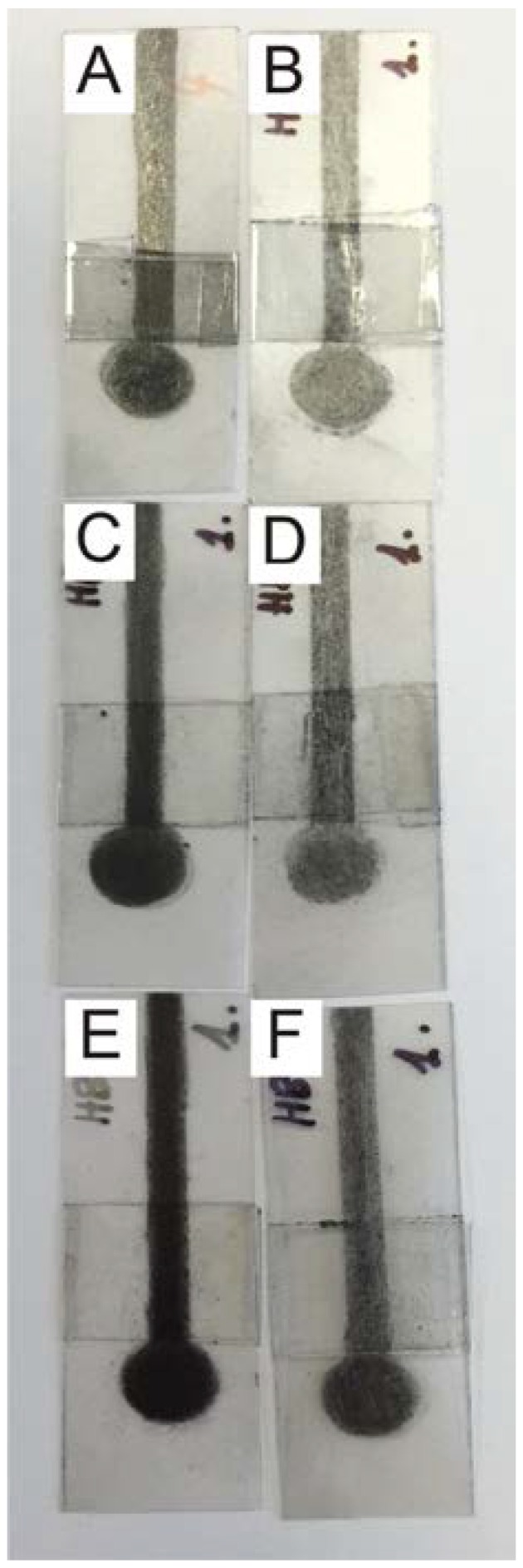
Optical images of PDEs fabricated using different batches of HB pencils, designated as PDE1 (**A**); PDE2 (**B**); PDE3 (**C**); PDE4 (**D**); PDE5 (**E**) and PDE6 (**F**), respectively.

Cyclic voltammetric measurements were performed using 1 mM hexaammineruthenium(III) chloride/0.1 M KCl and 1 mM potassium ferrocyanide (II)/0.1 M KCl redox probes. Figure S3 shows a series of cyclic voltammograms recorded for each PDE assayed, including the pencil-drawn working electrode PDE1 previously studied for comparative purposes. It is important to note that appropriate electrochemical measurements were only feasible when using PDE1, PDE3 and PDE5, even though very resistive voltammetric profiles and high peak-to-peak separation were observed for the aforementioned redox couples, particularly utilising PDE3 and PDE5. On the other hand, voltammograms of PDE2, PDE4 and PDE6 were not depicted in Figure S3 because the nature/composition of the graphite drawn on the plastic substrate (possibly influenced by the proportion graphite/clay in the leads used to create them) hindered the electrical connection of these PDEs to the electrochemical cell. In agreement to that, it was also observed experimentally during the drawing fabrication process of PDE2, PDE4 and PDE6 that these HB pencil leads’ were suspiciously breakable and softer than usually expected for this grade of writing pencils, not allowing the appropriate “dark’’ drawing onto plastic as otherwise exhibited by PDE1, PDE3 and PDE5 (Figure S4). Additional resistance measurements of different fabricated PDEs were also carried out (as described in the Materials and Methods Section). Referring to the values of resistances obtained (PDE1: 8 kΩ; PDE2: non measurable; PDE3: 22 kΩ; PDE4: non measurable; PDE5: 20 kΩ; PDE6: non measurable), which are strikingly different, it comes as no surprise that such PDEs fabricated using different HB pencils showed subsequently different behaviour towards the electrochemical probes assayed. The aforementioned differences observed in the electrochemical responses of PDEs demonstrated that an important lack of reproducibility detected in the properties of HB writing pencil leads’ decisively influences the effectiveness of the hand-made fabrication process of the sensors and their further feasible implementation as electrochemical platforms for sensing applications. Nevertheless, observing the electrochemical performance of PDE1, it was also demonstrated that it is possible to fabricate reproducible and sensitive electrochemical platforms if and when an adequate pencil was applied.

**Figure S5:**
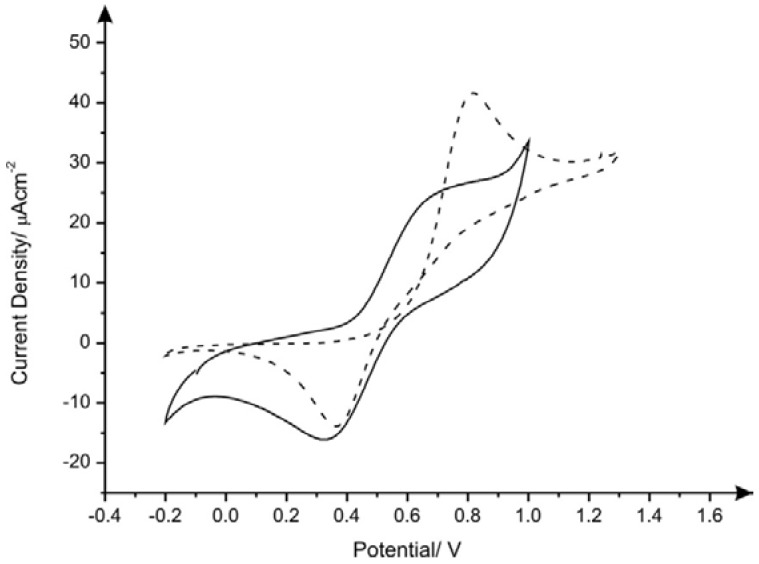
Comparison of cyclic voltammograms using PDE1 (solid line) and SPE (dotted line) recorded in 1 mM ammonium iron(II) sulphate/0.2 M HClO_4_. Scan rate: 5 mV·s^−1^.

**Figure S6:**
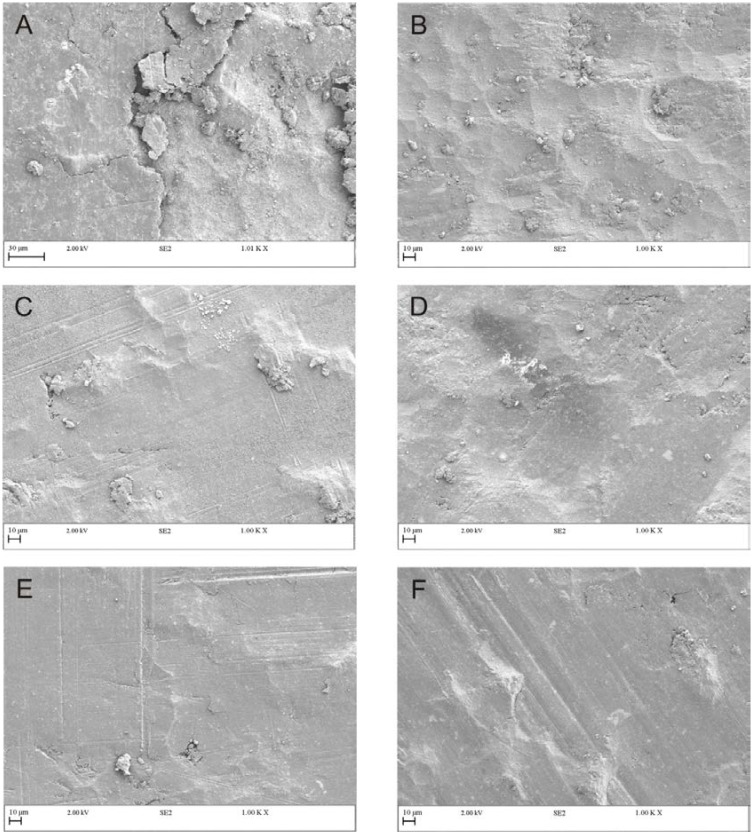
SEM images of different PDEs fabricated using different batches of HB pencils (boxes 1 to 6) on plastic substrates: PDE1 (**A**); PDE2 (**B**); PDE3 (**C**); PDE4 (**D**); PDE5 (**E**) and PDE6 (**F**), respectively.

Additionally, a comparative test between PDE1 and screen-printed electrodes (SPEs) was performed using the inner-sphere Fe^2+^/Fe^3+^ redox couple in order to evaluate differences in their surfaces. Cyclic voltammograms were recorded in 1 mM ammonium iron(II) sulphate in 0.2 M perchloric acid. This is a particular inner-sphere probe, which is known to be very sensitive to surface oxides and functional groups, especially carbonyl groups [31,32]. Figure S5 presents cyclic voltammetric profiles where the SPE exhibits unexpectedly larger peak-to-separation (434 mV) than PDE1 (305 mV). The improvement in voltammetric peaks using PDE1 in comparison to SPEs demonstrates that the different composition of PDEs influences the surface characteristics, which is likely a result of the introduction of oxygen or carbon-oxygen species coming from the clay component of the pencil’s lead. To explore the above results further, we gain insight from the physicochemical characterisation of our hand-drawn pencil electrodes. Here, we compare the surface morphology of the six fabricated PDEs utilised throughout this study, examining the SEM micrographs presented in Figure S6. There are clear differences between PDE1 and the other electrodes, where it can be seen that the surface is clearly rough and disordered (Figure S6A). It is also evident that dense graphitic flakes are present upon the surface of PDE1, which creates large defects that are likely directly responsible for the noticeably reactive electrochemical surface shown by this electrode (Figure S6A). Likewise, some graphitic deposits also appear in PDE3 and PDE5 (Figure S6C,E) providing some active sites on the surface, that could explain the electrochemical performance also reported for both PDEs. Alternatively, Figure S6B,D,F appear to exhibit smoother surfaces. As visualised on the SEM images, less material and likely lower quality graphite was consequently adhered after drawing onto the substrate. Striking lines observed on the micrographs reveal a higher proportion of binder/clay in the composition of the HB pencils used, which reasonably agrees with there being no electrochemical responses recorded when using PDE2, PDE4 and PDE6. It is clear that the pencils deposit different quantities and quality of graphite. This variation is then either due to the clay components or due to the quality of the graphite, so we look for further information through Raman and elemental analysis (EDX).

**Figure S7:**
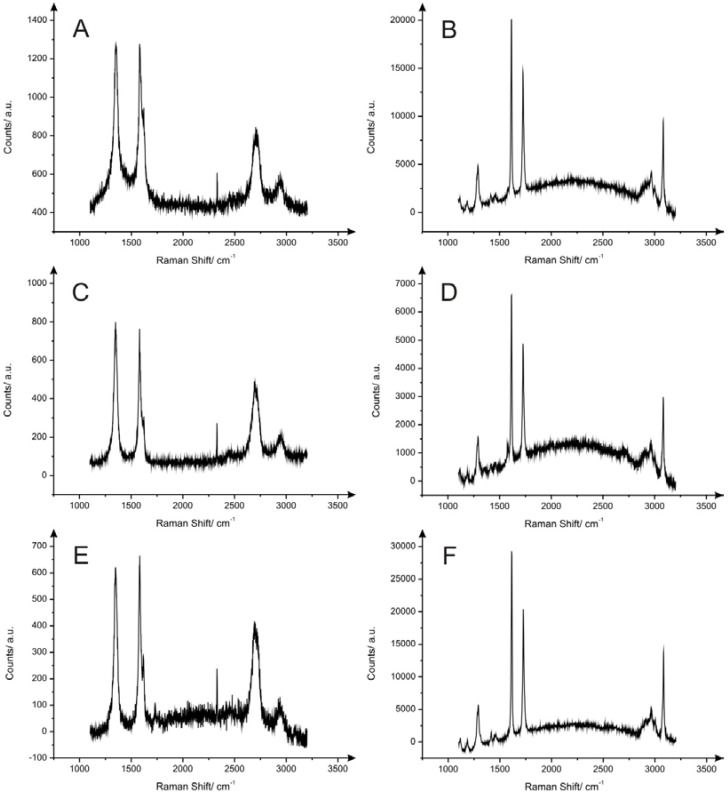
Raman results for PDEs (boxes 1–6) fabricated using different batches of HB pencils, designated as PDE1 (**A**); PDE2 (**B**); PDE3 (**C**); PDE4 (**D**); PDE5 (**E**) and PDE6 (**F**), respectively.

It is apparent through observation of Figure S7 that the results of the PDEs can be split into two groups. Analysis of Figure S7A,C,E, show the expected Raman signatures for graphite, with peaks at ca. 1400 (D peak), 1700 (G peak), and 2700 (2D peak) cm^–1^ [29,30]. The ratio of the G to 2D peaks in these spectra suggest graphite is present (but not bulk graphite, rather few layers given that that the 2D peaks are symmetrical). Through further analysis of the D peak, it is clear that these samples contain a larger number of edge plane-like sites/defects and oxygenated species content (supported by EDX and XPS data, see below) with the high/large intensity equal to that of the G peak, likely resulting in the beneficial electrochemical behaviour of these samples. The G/D peak ratio would suggest that graphene/graphite oxide is present, however, given that these peaks are not joined/merged and indeed can be fully de-convoluted, this suggests that high quality oxygenated graphite is present (or that of graphite with a large number of reactive edge plane like sites/defects). With respect to Figure S7B,D,F, these Raman spectra are clearly distinct from that previously interpreted above and thus it is evident that their quality of graphite is distinct. Although the samples have the presence of the D, G, and 2D bands noted previously, the peaks evident have clearly shifted in their positions and the intensity ratios recorded. Note the lack of a large 2D peak relative to the G peak, suggesting lower quality graphite present here than in the previous cases. Considering the intensity of the D band, these samples have a large content of oxygenated species (greater than that reported for graphene/graphite oxide, and the previous samples) and thus (agreeing with EDX and XPS) as a result are likely to be less conductive and electrochemically active (more insulating), causing interference with specific electrochemical reactions. Finally, given the shift and contributions in the Raman spectra coming through from the background support in the latter cases, it is clear that less graphitic material has been deposited. Considering all of the above stated analysis, lower quality graphite is deposited in such instances, leading to the unfavourable electrochemical responses observed.

Supporting EDX analysis is presented in Table S1. Analysis of Group 1, which includes PDE1, PDE3, and PDE5, and Group 2, which corresponds to PDE2, PDE4, and PDE6, pencil leads appear to follow a similar trend as previously stated. Group 2 PDEs exhibit a larger contribution from oxygenated species than that of Group 1, which may influence their integration with the substrate surface and have implications for surface adhesion. Further to this, the larger number of oxygenated species (if not the correct species desired for a specific target analyte) will limit the interaction of the electrode surface and the analyte or redox species in solution. Therefore, the Group 2 PDEs are likely to be less conductive with respect to their high oxygen content. In fact, non-measurable resistances of these PDEs, as reported above, proves that these electrodes will not be suitable for electrochemical utilisation. Regarding the presence of Si and Al, also detected by EDX (Table S1), which indicate the contribution of clay component of the pencils’ leads, higher amounts of these species were observed on PDEs from Group 1, meaning increased adhesion of the material after drawing onto the plastic substrate. This observation is clearly in agreement with the conductivity exhibited from this group of electrodes and demonstrates that the pencil grades are effective for their electrochemical application. Further consideration is given to the presence of Fe. It is present in all Group 1 samples where electrochemistry is able to be recorded/performed. It is possible that this contaminant is an electrocatalytic component and is causing the electrochemical behaviour observed herein.

We next analyse the PDEs via XPS to explore the surface characteristics of each of the electrode platforms. De-convolution of the XPS presented in Table S2 reveals that there has been a clear deposition of graphite upon the substrate, however, there are variances within the percentages of all the compounds present on the surface. Further analysis of the O/C ratios for each of the PDEs (1–6) correspond to the following values 0.087, 0.091, 0.018, 0.058, 0.026, 0.043, respectively. It is clear that the deviation within these values is low and one would expect to see similar electrochemical behaviour when utilising a surface orientated probe. As stated previously, PDE 1, PDE 3 and PDE 5 are the only fabricated electrochemically active sensors.

In summary, due to the variation within pencil leads from a range of boxes, the utilisation of these PDEs is considered to be highly unreproducible, as the overall electronic structure and surface-active groups available can vary dramatically from box-to-box. 

### 3.3. Application of PDEs for the Determination of Dopamine and Acetaminophen: Comparison with Screen-Printed Electrodes (SPEs)

We now explore the applicability of PDEs to detect typical electroactive molecules in order to demonstrate their suitability for sensing purposes. With respect to the aforementioned issues reported in this study, concerning the observed lack of reproducibility of the PDEs fabricated using HB pencils from different batches and the performance of HB in comparison with other grades of pencil, the working electrode denoted as PDE1 elsewhere has been utilised for the determination of dopamine and acetaminophen.

Based on successful preliminary inspections of voltammetric peaks recorded for 0.1 mM of dopamine and acetaminophen in 0.1 M pH 7.4 PBS/0.1 M KCl using a PDE fabricated when drawing with HB pencil from Box 1, the electroanalytical performance of an optimised PDE (drawn 60 times) was next assessed towards the direct determination of both analytes. Calibration curves were constructed for dopamine and acetaminophen separately in 0.1 M pH 7.4 PBS/0.1 M KCl using CV data and a single PDE over the entire concentration range (5–120 µM). Three replicates for each concentration were performed.

**Figure 3:**
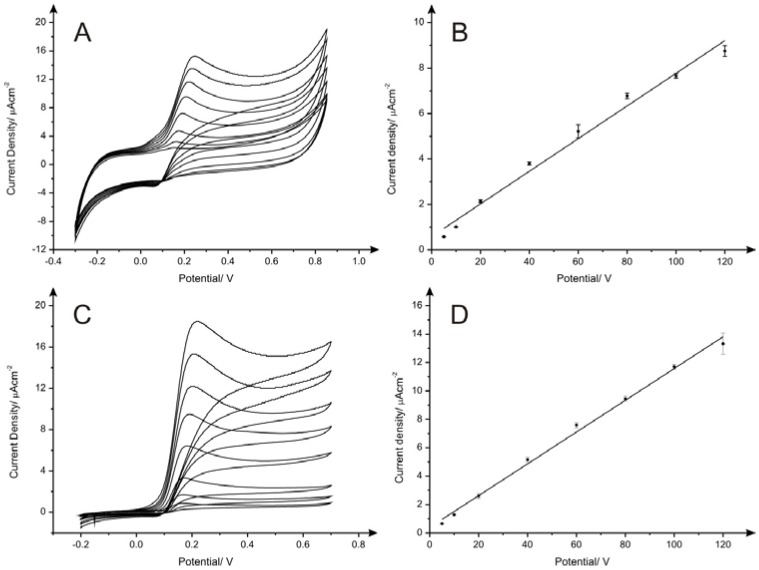
Cyclic voltammograms and calibration curves of dopamine using PDE1 drawn 60 times with HB pencil from Box 1 (**A**) and (**B**) and screen-printed electrodes (**C**) and (**D**) in 0.1 M pH 7.4 PBS/0.1 M KCl. Each data point shown in (**B**) and (**D**) is the average and standard deviation of the replicates (*N* = 3). Scan rate: 5 mV·s^−1^.

**Figure 4:**
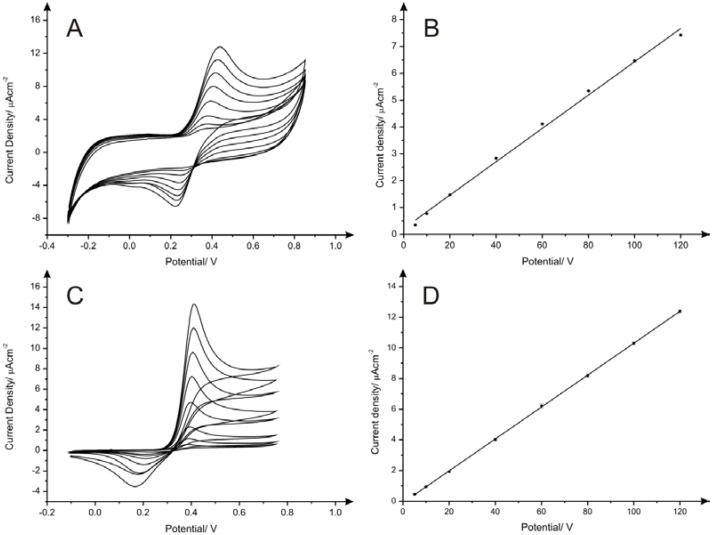
Cyclic voltammograms and calibration curves of acetaminophen using PDE1 drawn 60 times with HB pencil from Box 1 (**A**) and (**B**) and screen-printed electrodes (**C**) and (**D**) in 0.1 M pH 7.4 PBS/0.1 M KCl Each data point shown in (**B**) and (**D**) is the average and standard deviation of the replicates (*N* = 3). Scan rate: 5 mV·s^−1^.

As represented in Figure 3A,B, the electroanalytical peak observed for dopamine at*ca*. +0.19 V increases linearly as a function of the concentration (*I*/µA·cm^−2^ = 0.072 µA·cm^−2^ µM^−1^ + 0.580 µA·cm^−2^; *R*^2^ = 0.987), with an limit of detection (3σ) found to correspond to 5.27 µM. Also, a very good linear response was observed for the oxidation peak of acetaminophen (Figure 4A,B) at ca. +0.39 V over the analytical range studied (*I*/µA·cm^−2^ = 0.062 µA·cm^−2^ µM^−1^ + 0.220 µA·cm^−2^; *R*^2^ = 0.997) providing a limit of detection of 2.61 µM. It is interesting to note that the oxidation potentials of both analytes using PDEs undergo a positive potential shift upon an increased current. However, as depicted in Figure 3C and Figure 4C, the oxidation potential values are very similar to the peak potentials observed upon the utilisation of SPEs for the determination of the same analytes, which corresponds to +0.18 V and +0.40 V for dopamine and acetaminophen respectively. This indicates that in terms of electron transfer rate kinetics PDEs do not exhibit noticeable differences in comparison with SPEs, which is unexpected at first according to the different nature of the graphite used for the fabrication of PDEs and SPEs and the different creation processes.

Calibration curves obtained in 0.1 M pH 7.4 PBS/0.1 M KCl for dopamine and acetaminophen using SPEs are shown in Figure 3D and Figure 4D, respectively. An excellent linear response was observed for the oxidation of the analytes over the concentration range studied (Dopamine: *I*/µA·cm^−2^ = 0.112 µA·cm^−2^·µM^−1^ + 0.387 µA·cm^−2^; *R*^2^ = 0.996; Acetaminophen: *I*/µA·cm^−2^= 0.104 µA·cm^−2^·µM^−1^ – 0.105 µA·cm^−2^; *R*^2^ = 0.999). The limits of detection achieved using SPEs were 3.76 µM and 0.84 µM for dopamine and acetaminophen respectively, which is in agreement with data previously reported [26]. In terms of sensitivity, the reported PDEs (drawn 60 times) demonstrate their suitability for analytical applications. Additionally, low cost fabrication and easy to use production could make them a promising alternative for the development of a new generation of handmade electrodes. However, as we have demonstrated in this paper, the lack of reproducibility in the composition of pencils used for the fabrication is an important drawback that determines decisively their feasible implementation as disposable sensors and therefore PDEs are *not* considered at this point as a competitive alternative to well-known screen-printed electrodes.

**Figure S8:**
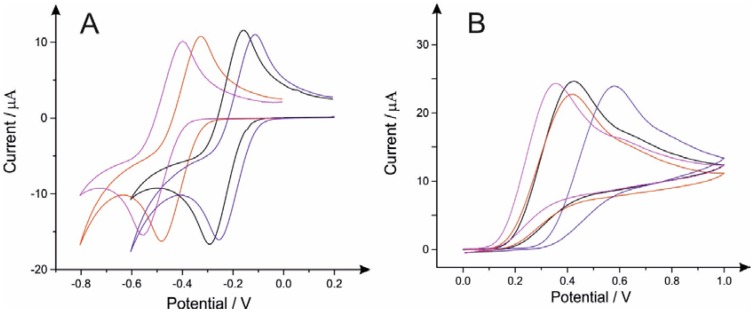
Typical cyclic voltammograms recorded using PDE1 (drawn 60 times) towards 1 mM hexaammineruthenium(III) chloride/0.1 M KCl (**A**) and 1 mM uric acid/0.1 M PBS (**B**), utilising screen-printed Ag/AgCl (black line), screen-printed carbon (red line), saturated calomel electrode (purple line) and pencil-drawn graphite references (pink line). Scan rate: 50 mV·s^−1^.

Last, we compare the effect of pseudo pencil-drawn reference electrodes with that of a screen-printed Ag/AgCl alternative. Such studies are presented as many reports within the literature utilise this pencil-drawn fabrication method to provide low cost and simplistic sensors. Depicted in Figure S8A are comparative voltammograms within hexaammineruthenium (III) chloride, using pencil-drawn, screen-printed graphitic, screen-printed Ag/AgCl and benchmark SCE reference electrodes. Inspection of the cyclic voltammograms indicates that each of the systems utilised herein offer comparative peak-to-peak separations, however, analysis of the corresponding formal potentials (*E*^0^) (within Table S3) for each configuration indicates that the SCE and screen-printed Ag/AgCl references demonstrate the lowest values, −0.18 V and −0.22 V, respectively. Also presented in Figure S8B is a comparison of each of these reference electrodes with the common electrooxidation of uric acid. It is clear that all the pseudo references used herein are comparable and exhibit lower peak potentials than that of the SCE.

## 4. Conclusions

The electrochemical and physicochemical characterisation of these hand-drawn pencil electrodes (PDEs) on ultra-flexible polyester substrates was explored in terms of the number of draws applied for their fabrication, the grade of pencils used, and the different batches assayed.

The favourable electron transfer kinetic achieved by applying 60 systematic drawings for the fabrication of PDEs was employed in the whole study. Unexpectedly, the commercial HB grade pencils, which present a lower graphite composition in comparison to other higher grades, exhibited better electrochemical properties with respect to the presence of oxygenated species upon fabrication of the drawn PDEs towards several inner- and outer-sphere redox probes than other grades utilised. The higher level of edge plane defects and dense graphitic deposits generated on the surface of these PDEs improved the overall adhesion of the material to the substrate and consequently contributed to the enhancement of their electrochemical properties.

Unfortunately, a substantial lack of reproducibility in the PDEs fabricated using different batches was demonstrated. PDEs fabricated when employing six different batches of HB pencils were thoroughly tested and striking differences in the surface characteristics and electrochemical performances were unexpectedly found. Raman, EDX, SEM and XPS analyses revealed different composition of the HB pencil leads from diverse batches, and clear visual differences in both quality of graphite and its adhesion to the substrate were reported, demonstrating a dramatic influence on the electrochemical behaviour of resulting PDEs. Nevertheless, the unique PDE that exhibited outstanding surface properties and subsequently exceptional electrochemical behaviour was successfully used for the electrochemical sensing/detection of dopamine and acetaminophen.

Far from being a promising alternative to SPEs, due to their questionable reproducibility and repeatability, strong dependence on graphite composition and difficult mass production, PDEs have showed suitability for electrochemical applications according to the good limit of detection achieved for the target analytes. However, this approach has demonstrated that PDEs are not feasible as disposable electrodes in their current state, and do not represent a competitive alternative to superior screen-printed platforms. We reiterate that these SPEs can be mass-produced and as such have a large economy of scale; for instance, one standard PDE (utilised herein) has a lead-time of 30 min. However, to create a batch of fully screen-printed electrodes (i.e. graphite, reference and dielectric layer), it will take 4 min per electrode. 

## Figures and Tables

**Table 1:** Overview of current literature on pencil-drawn electrode systems, in order of publication date.

Electrodes Fabricated	Pencil and Substrate Utilised	Target Analytes	Analytical Method	Reference
Pencil-drawn macroelectrode	Derwent, Staedtler Mars Lumograph, FILA and Koh-i-Noor Hardtmuth (HB, B, 2B, 3B, 4B, 6B, 8B explored) upon paper substrates	Potassium ferrocyanide, ascorbic acid and sunset yellow	Thin-layer chromatography with amperometric detection and cyclic voltammetry	[12]
Pencil-drawn dual electrode with pseudo reference electrode	Staedtler Mars (grade “3B” only) upon paper substrates.	Ascorbic acid, dopamine and paracetamol	Thin-layer chromatography with amperometric detection and cyclic voltammetry	[13]
Pencil-drawn working macroelectrode	Staedtler Mars (grade “3B” only) upon paper substrates	Potassium ferrocyanide and 1,2-hydroxybenzene	Cyclic voltammetry	[14]
Pencil-drawn counter electrode only	Bulk pencil “lead’’ working electrode with the counter electrode drawn using Pental (6B grade only) pencil upon paper substrates	*p*-nitrophenol	Differential pulse voltammetry	[15]
Pencil-drawn immune device	6B-type Black Pencil only upon a paper substrate	Carbohydrate antigen 199	Electro-chemiluminescence	[16]
Pencil-drawn strain gauges and chemiresistor	Blick pencils (9H, 2H, HB, 2B, 6B, 9B explored) upon paper substrates	Toluene, THF, ethyl acetate, methanol, hexane and acetone	Resistance measurements	[10]
Pencil-drawn working macroelectrode with pseudo reference and counter electrode also drawn	Working electrode was a bespoke “pencil’’ manufactured utilising a mixture of carbon powder, sodium bentonite and potassium silicate, then doped with decamethylferrocene or cobalt(II) phthalocyanine and drawn upon paper substrates	Cysteine and hydrogen peroxide	Linear sweep voltammetry and cyclic voltammetry	[17]
Pencil-drawn working macroelectrode	Derwent (grade 6B only) upon polyvinyl chloride substrate	Lead (II)	Anodic stripping voltammetry	[18]
Pencil-drawn working macroelectrode with pseudo reference and counter electrode	Working electrode was a “pencil’’ manufactured using a mixture of carbon powder, sodium bentonite and potassium silicate. Ag/AgCl doped pencils leads were used for drawing reference electrode. Chromatographic paper as substrate	Ortho-diphenols in extra virgin olive oil and sunflower oil	Cyclic voltammetry	[19]
Pencil-drawn electrodes attached to poly(methyl methacrylate) (PMMA) electrophoresis chips	Pencil grade is not specified. Drawn upon chromatographic paper platform	K^+^, Na^+^ and Li^+^	Electrical conductivity	[1]
Fully-drawn pencil sensor	Staedtler Mars 4B, 5B, 6B and 9B grades’ pencils for drawing working, counter and reference electrodes upon paper substrate	Dopamine	Cyclic voltammetry	[20]
Fully-drawn origami paper analytical device	Staedtler Mars 6B pencil was used for drawing working, counter and reference electrodes on paper substrate	Glucose	Cyclic voltammetry	[21]
Pencil-drawn working macroelectrode	Commercially available Staedtler Mars tradition pencils upon an ultra-flexible polyester substrate (2H, H, HB, B, 2B, 3B, 4B, 5B, 6B) explored; 10 draws	Hexaammineruthenium(III) chloride, ammonium iron(II) sulfate, potassium ferricyanide, *p*-benzoquinone and simultaneous detection of lead(II) and cadmium(II) ions	Cyclic voltammetry and anodic stripping voltammetry	[11]
Pencil-drawn working and reference macroelectrodes	Commercially available Derwent pencils upon an ultra-flexible polyester substrate (HB, B, 2B, 3B, 4B 7B, 8B, 9B explored); 60 draws. Reference electrodes have been drawn with a HB and compared to screen-printed alternatives	Hexaammineruthenium(III) chloride, potassium ferrocyanide, ammonium iron(II) sulfate, dopamine and acetaminophen	Cyclic voltammetry	This Work

## Supplementary Materials

**Table S1:** EDX analysis of PDEs (1 to 6) fabricated using different batches (boxes 1–6) of HB pencils.

Samples	Elemental Analysis/%wt.				
	C	O	Si	Al	Fe
**PDE1**	66.83	17.82	7.19	5.50	1.07
	67.22	19.21	6.40	4.86	1.11
	67.09	16.33	7.91	5.95	1.20
**PDE2**	63.49	32.52	2.02	1.97	
	62.61	32.80	2.17	2.41	
	63.18	30.49	3.26	3.07	
**PDE3**	66.39	19.19	7.21	5.31	1.03
	66.81	20.20	6.48	4.79	0.95
	66.70	18.58	7.45	5.44	0.96
**PDE4**	60.70	26.69	6.19	5.79	
	62.19	28.83	4.38	4.04	
	61.90	28.73	4.68	4.22	
**PDE5**	66.81	17.87	7.82	5.68	0.98
	67.64	20.98	5.68	4.35	0.84
	67.09	22.71	5.12	3.87	0.73
**PDE6**	62.01	26.03	5.51	4.85	0.88
	63.64	25.54	5.01	4.53	0.79
	62.77	27.11	4.56	4.35	0.69

**Table S2:** XPS analysis of the fabricated PDEs using HB pencils from different boxes (1–6).

Element	Atom %					
	PDE1	PDE2	PDE3	PDE4	PDE5	PDE6
C 1s	81.65	74.82	94.84	80.97	93.94	87.55
O 1s	12.99	18.51	3.90	13.97	4.71	9.30
Si 2p	1.97	3.66	0.65	2.89	0.74	1.69
Al 2p	1.27	2.95	0.61	2.20	0.56	1.41

**Table S3:** Analysis of the formal potentials using a range of reference electrodes, using 1 mM hexaammineruthenium(III) chloride / 0.1 M KCl. Scan rate: 50 mV·s^−1^.

Type of Reference Electrode	Formal Potential / V
Screen-Printed Graphite	+0.36
Screen-Printed Ag/AgCl	−0.22
Saturated Calomel Electrode	−0.19
PDEs	−0.40
